# Exploring the nature, development and effects of complex emotions in Sheffield and Plymouth, UK: a longitudinal mixed methods study protocol

**DOI:** 10.1136/bmjopen-2026-117088

**Published:** 2026-10-26

**Authors:** Sally Ohlsen, Dion Anderson, Anastasios Bastounis, Kamaldeep Bhui, Andrew Booth, Christopher D Burton, Richard Byng, Timothy James Chico, Lisa Couperthwaite, Helen Crimlisk, Tim Dalgleish, Michael Doyle, Saffron Gravestock, Rebecca Hardwick, Michelle Horspool, Katie Hullock, Tim Kendall, Joanna Leaviss, Tiffany Markwick, Venet Osmani, Vanessa Pinfold, Molly Robinson, Georgia Jean Sullivan, Anthea Sutton, Felicity Thomas, Elissa Rosina Thompson, Evette Wilson, Scott Weich

**Affiliations:** 1 SCHARR, The University of Sheffield, Sheffield, South Yorkshire, UK; 2 Complex Emotions Lived Experience Advisory Panel, Sheffield, UK; 3 Division of Population Health, School of Medicine and Population Health, The University of Sheffield, Sheffield, UK; 4 Psychiatry, University of Oxford, Oxford, UK; 5 Academic Unit of Primary Medical Care, University of Sheffield, Sheffield, UK; 6 Centre for Clinical Trials and Health Research, University of Plymouth, Plymouth, UK; 7 Department of Infection, Immunity and Cardiovascular Disease, The Medical School, The University of Sheffield, Sheffield, South Yorkshire, UK; 8 The McPin Foundation, London, UK; 9 Sheffield Health Partnership University NHS Foundation Trust, Sheffield, UK; 10 University of Cambridge, Cambridge, UK; 11 SHHS, University of Huddersfield, Huddersfield, UK; 12 Medical School, University of Exeter, Exeter, UK; 13 The University of Sheffield, Sheffield, UK; 14 Mental Health, NHS England, Redditch, UK; 15 University of Sheffield, The University of Sheffield, Sheffield, UK; 16 Queen Mary University of London, London, UK; 17 McPin Foundation, London, UK; 18 University of Exeter, Exeter, UK

**Keywords:** MENTAL HEALTH, QUALITATIVE RESEARCH, Longitudinal studies

## Abstract

**Abstract:**

**Introduction:**

Complex emotions include difficulties with emotional regulation, impulsivity, identity, relationships and functioning. These experiences are often construed as borderline personality disorder, a highly stigmatised diagnosis. We will use a transdiagnostic approach to investigate the nature, course and lived experience of complex emotions, with lived experience involvement embedded throughout the programme.

We aim to (1) model symptom and functional trajectories to identify subgroups with distinct needs; (2) elucidate mechanisms underlying outcomes, including interactions with social, cultural and environmental factors; (3) investigate associations between complex emotions and physiological and behavioural measures; and (4) integrate findings to inform interventions and service delivery.

**Methods and analysis:**

We will undertake a mixed methods longitudinal study co-developed with our Lived Experience Advisory Panel (LEAP) and informed by evidence synthesis. We aim to recruit 300 participants who self-identify as experiencing complex emotions through health services, third-sector organisations and probation services, with baseline assessments and 6 monthly follow-up. Two nested substudies will comprise (1) longitudinal biographical interviews and (2) ecological momentary assessment (EMA) of experiential, physiological and behavioural data. Quantitative data will be analysed using multivariate and longitudinal trajectory modelling methods to identify underlying dimensions and phenotypic subgroups. Qualitative data will undergo reflexive thematic and narrative approaches to identify shared experiences, coping strategies and meaning-making. EMA data will be analysed using network and time-series approaches to examine within-person and between-person associations and temporal instability. Findings will be integrated to develop an overall understanding of the nature, trajectories and mechanisms of complex emotions.

**Ethics and dissemination:**

The cohort and qualitative studies were approved by the East Midlands (Derby) National Health Service (NHS) Research Ethics Committee (REC) and the Health Research Authority (HRA) (IRAS 353789, REC 25/EM/0128; 4 July 2025). The EMA substudy was approved by Cambridge East NHS REC and HRA (IRAS 364085, REC 26/EE/0171; 11 August 2026). Dissemination strategies will be co-developed by researchers, LEAP members and lived experience co-investigators. All partners will contribute to peer-reviewed publications, conference presentations and accessible outputs. The anticipated date of study completion is 31 March 2029.

**STRENGTHS AND LIMITATIONS OF THIS STUDY**

- Strong lived experience involvement across study design, delivery, analysis and dissemination.
- Transdiagnostic approach with inclusive recruitment across healthcare and community settings.
- Mixed methods, longitudinal design combining evidence synthesis, cohort, qualitative and ecological momentary assessment data.
- Discovery-focused, iterative approach to data collection and analysis.
- Recruitment limited to two geographical sites for pragmatic reasons.

## Introduction

 Complex emotions include difficulty with emotional regulation, impulsivity, identity disturbance, unstable relationships, self-harm, dissociation, brief psychotic episodes and suicidality.^1^ They cause distress and impaired functioning and are associated with poor physical health, including obesity, cardiovascular disease and epilepsy.^2–4^ People presenting with these difficulties are most often diagnosed with borderline personality disorder (BPD),^5–7^ but the validity of which has been questioned^5 7 8^ partly because of extensive comorbidity with depression, anxiety, post-traumatic stress disorder (PTSD), bipolar disorder, eating disorders and autism and attention-deficit hyperactivity disorder (ADHD).^4 9–11^

Stigmatisation of those diagnosed with BPD is particularly problematic.^12 13^ Many healthcare professionals and the general public hold negative perceptions that conflate BPD with irresponsibility, manipulation and unpredictability.^14 15^ People with the diagnosis report feeling misunderstood, dismissed or labelled as manipulative,^16 17^ hindering access to appropriate care.^7 14 18 19^

We therefore use different language and a transdiagnostic approach to study complex emotions and interactions between symptoms, neurobiology and socio-environmental factors.^20 21^ This approach has been coproduced with lived experience partners and aims to enable research that extends beyond clinical settings. One challenge, however, is that most knowledge about complex emotions derives from studies of BPD, based on diagnostically-defined samples.^22^ It is unclear how representative these are of the wider population of people who experience complex emotions but do not seek or are excluded from care.

Complex emotions arise from interactions between genetic, environmental and social factors.^1^ Childhood trauma, particularly abuse or neglect, is a strong predictor,^23 24^ while heritability is estimated at 40%.^25^ Neurobiological studies implicate dysfunction in emotion-regulating brain regions,^26^ while psychological theories emphasise early attachment and difficulties with social cognition.^27^ Cultural factors also shape how symptom expression and interpretation.^28^ Symptoms associated with the diagnosis of BPD usually begin in adolescence,^29^ fluctuate over time and often continue over the life course.^30^

Evidence of effective interventions is mostly limited to BPD research. National Institute for Health and Care Excellence guidance discourages pharmacological treatment^31^ and advises against brief psychological therapy. Specialist services were recommended in the UK^32^ but many pilots were decommissioned despite moderate evidence that psychological approaches reduce BPD symptoms and improve functioning.^33^ Over 90% of people with BPD are prescribed psychotropic drugs,^34–37^ despite sparse evidence of effectiveness and no licensed treatment for this indication.^38^

The Complex Emotions Hub, one of six hubs supported by the UKRI Mental Health Platform, will move beyond diagnostic labels and include people whose voices are under-represented in research. Lived experience perspectives will be embedded throughout the research, to ensure this is meaningful, inclusive and ethical.^39^

### Aims

We will investigate the nature, course and lived experience of complex emotions and elucidate the mechanisms through which biological, psychological and social factors shape outcomes, using a transdiagnostic approach.

### Research questions

1. How should complex emotions be conceptualised, considering the nature, distribution, severity and trajectory of symptoms and functional outcomes?
2. Are there meaningful and stable subgroups of people who experience complex emotions, based on symptomatic and functional outcomes?
3. How do social and cultural factors influence experiences and outcomes associated with complex emotions, and how might we integrate this evidence with biological and psychological models?
4. How do features associated with complex emotions interact to trigger emotional crises and problematic impulsive behaviours, particularly self-injury and what coping strategies might prevent this?

## Objectives

We will:

- Recruit and support a diverse cohort of 300 individuals with lived experience of complex emotions from health services and community settings, including probation, homeless and drug and alcohol services.
- Collect longitudinal data on symptoms, social functioning and social and environmental circumstances.
- Collect in-depth qualitative data on lived experience and interplay between complex emotions and social and cultural environments.
- Make anonymised data available securely through collaboration with DATAMIND for consented participants, supporting further research.
- Integrate study findings to generate insights to inform interventions and service delivery.

### Patient and public involvement statement

This study was developed together with people with lived experience of complex emotions. Three lived experience experts were coapplicants on the research funding application. Colleagues from the McPin Foundation, including a lived experience coapplicant, helped develop the study, combining lived experience and methodological expertise.

A 12-member Lived Experience Advisory Panel (LEAP) was established to bring diverse perspectives on complex emotions. LEAP members work collaboratively with the research team across all stages of the study, shaping the research and study procedures, co-developing participant materials, contributing to research ethics applications and influencing choice of baseline measures and qualitative interview design. They also contribute to recruitment planning, data interpretation and dissemination, including coauthoring publications and cocreating accessible outputs for participants, practitioners and communities.

LEAP members meet at least monthly as a full team or in subgroups focused on specific themes or study components and decisions are made collectively. LEAP members are involved in key strategic decisions regarding collaboration with industry partners. Early outputs included a coproduced Ways of Working charter and Hub authorship policy. Our approach emphasises healthy, communicative relationships and sustaining a strong LEAP.

LEAP members and lived experience co-investigators can take on peer researcher roles, supported by tailored training and ongoing supervision. All team members, including lived experience partners, have access to regular debriefing and emotional support, recognising the sensitive nature of the research.

## Methods and analysis

### Study design

We will undertake a longitudinal, multicentre, mixed methods study, supported by evidence synthesis and two nested substudies, designed to examine experiences, symptoms and outcomes of living with complex emotions.

### Evidence synthesis

Evidence synthesis will address uncertainties and information needs across the programme. We will synthesise evidence from aetiological and interventional research, longitudinal studies and qualitative research to examine mechanisms underlying the development and outcomes of complex emotions. This will incorporate current frameworks, conceptualisations and phenotypes and inform development of a preliminary system map, including recursive loops linking causal factors, complex emotions and outcomes.

A formal realist review exploring the causes and mechanisms of complex emotions will be conducted, with the full protocol registered on PROSPERO. The review will employ an iterative, three-staged approach to searching, comprising: mining of theory terms and combining population terms with theory keywords, followed by expanding and searching full texts and citations if retrieval is sparse. PsycINFO searches conducted for stage one will be translated in stage two across Ovid MEDLINE, Scopus, CINAHL via EBSCO and PTSDpubs via ProQuest. Stage three will involve backward and forward citation chaining on included studies using the Citations, Lead authors, Unpublished materials, Google Scholar, Theories, Early examples, and Related projects (CLUSTER) searching framework.^40^ We will not have a date limit on searches, which will extend from database inception until search completion, by the end of 2026.

Eligibility criteria will be based on a PCO framework:

*Population:* Populations with complex emotions, including BPD. Population terms will be broadened to include terms relating to complex PTSD (cPTSD) and emotional dysregulation. Excluded populations will include acute PTSD and studies of general personality disorders where it is not possible to separate data for BPD or relevant populations.

*Context:* The social, developmental, interpersonal and environmental circumstances in which theories propose that mechanisms contributing to complex emotions operate.

*Outcomes*: Complex emotional experiences, symptoms, functioning and other outcomes associated with the hypothesised mechanisms.

Records will be screened and extracted by a team of reviewers, with 20% independently double screened to assess consistency with eligibility criteria. Data relating to Context–Mechanism–Outcome Configurations (CMOCs) will be cross-checked for accuracy. We will assess the rigour, relevance and richness of the evidence using established realist review approaches, prioritising richness where multiple studies of the same theory are identified.

Programme theories will be developed and refined iteratively in consultation with key stakeholders, including LEAP members and clinical experts. Refined theories will be presented as a series of CMOCs describing explanations for the causes and mechanisms of complex emotions.

Findings will be presented using tables, figures and visualisations, including a system map derived from the developing programme theories to provide an overview of symptoms and phenotypes associated with complex emotions, their characteristics and supporting evidence. We will examine evidence linking symptoms and symptom clusters with one another, social and interpersonal environments, causal antecedents and outcomes.

### Cohort study

#### Setting

We will recruit participants from Sheffield (an industrial city in northern England) and Plymouth (a coastal city in Southwest England) and surrounding areas. Sites were chosen for their mix of urban and rural populations and varying deprivation. Limiting recruitment to these areas ensures close collaboration with trusted partners and allows for in-person interviews and follow-up support where needed. Localised recruitment will also reduce the risk of fraudulent participation (see below).

Recruitment will occur through National Health Service (NHS) services (primary and secondary care), probation services, community organisations (including voluntary sector partners) and peer networks, using comparable strategies across sites. To promote diversity and inclusion, we will undertake targeted outreach through local and community-based organisations with links to groups typically under-represented in research, including those who may not engage with mainstream mental health services. Participants will be asked where they heard about the research at screening and in more detail at baseline interview.

#### Participants and recruitment

Participants do not need to identify as having a mental health diagnosis or to have accessed treatment or support. We will work with community partners and grassroots groups to reach individuals often excluded from research.^41^

#### Incentives

Participants will receive non-exchangeable shopping vouchers to acknowledge their time and contribution as evidence suggests that this improves participation in survey-based research^42^ and represents an ethically justifiable way to recognise participants’ time, expertise and emotional labour.^43 44^ Incentives will be provided promptly after each stage: £30 following baseline interview and questionnaires, £10 for each set of follow-up self-report measures (up to five) and £25 for each qualitative substudy interview. For the ecological momentary assessment (EMA) substudy, participants will receive £25 for enrolling and completing onboarding, £25 for completing 50% of the questions and £20 for participating in the report-assisted interview. Smartwatches will be provided and returned following data collection.

To reduce fraudulent participation, IP addresses will be checked as an indicator of UK and Sheffield/Plymouth location. Participants may participate while away from home or using a VPN, with these circumstances confirmed by the study team. Baseline interviews must be completed in person or by video. Any remaining concerns will be reviewed by the study team.

#### Inclusion criteria

Participants must:

- Be aged ≥16 years.
- Live, work or study in the Sheffield or Plymouth recruitment areas. Participants who move outside of this area will be eligible to continue.
- Self-identify as experiencing difficulties associated with complex emotions such as emotional dysregulation, difficulties with identity or relationships, harmful impulsive behaviours (including self-harm) or other behaviours that feel out of control.
- Be able to complete the study assessments in English.
- Score above the threshold on both screening questionnaires (see below).

#### Exclusion criteria

Participants will be excluded if they:

- Are aged <16 years.
- Live, work or study outside the recruitment area.
- Score below the threshold on either screening questionnaire.
- Cannot complete the study assessments in English.
- Cannot provide informed written or verbal consent.

Exclusion of those unable to complete assessments in English is a study limitation, reflecting the absence of validated translations of many study measures.

#### Screening

Potential participants will be invited to complete online screening questions to ensure they meet the inclusion criteria. The screening questionnaire will be delivered via online survey (paper screening and prepaid return envelopes will be provided on request). We will use broad criteria to meet our research aims by adopting a lower scoring threshold than normally used in studies of BPD. Cut-off scores were chosen pragmatically to ensure inclusion of those who experience complex emotions but who might not meet diagnostic criteria for BPD or related disorders. The screening questionnaire will take approximately 10 min to complete.

We will use the following self-report questionnaires:

- McLean Screening Instrument for BPD,^45 46^ a 10-item self-report measure for which the recommended clinical scoring threshold is 7. We will use a cut-off score of 4 to include those experiencing complex emotions without meeting diagnostic criteria.
- The 12-item Personality Functioning Scale-Brief Form 2.0,^47 48^ a brief measure of personal and interpersonal functioning. A score of 31 or above indicates clinical dysfunction. We will use a cut-off of 25 to capture lesser (but significant) functional impairment.

Eligible participants will be those scoring above the thresholds on both measures.

#### Baseline assessment

Interviews will be conducted online or in person by trained researchers. Interviewers will not be blind to screening data. Interviews will include:

- Structured Clinical Interview for Diagnostic and Statistical Manual of Mental Disorders (DSM)-5 Personality Disorders (SCID-5-PD).^49^
- Questions about demographics, physical health, mental health history, service use and social factors.

#### Interviewer training, supervision and inter-rater reliability

Interviewers will receive training in administering and scoring the SCID-5-PD BPD section, including supervised practice interviews and discussion of discrepant or ambiguous cases. Interviewers will be supported by an experienced clinician researcher with expertise in personality disorder assessment and use of this instrument.

Inter-rater reliability will be assessed at an early stage using independently rated interviews. A subset of interviews will be audio-recorded (with consent) and independently scored by a second trained interviewer blind to the first interviewer’s ratings. Agreement on BPD criteria and scoring will be assessed and discrepancies reviewed, with further training provided as required.

Participants will be asked to complete additional validated self-report questionnaires at baseline, capturing complex emotions symptoms, quality of life, trauma, relationships, social circumstances and service use. These include measures selected with the LEAP. As there were no validated measures of positive character strengths, we co-developed one with our lived experience partners and will validate this over the course of our research. Measures were selected to harmonise data across the UKRI Mental Health Platform. A full list of measures is shown in table 1.

**Table 1:** Cohort study data collection schedule

	Data collection points							
	Baseline			Month 6	Month 12	Month 18	Month 24	Month 30
Measure	Screen	Interview	Self-report measures (SRM 1)	Self-report measures (SRM 2)	Self-report measures (SRM 3)	Self-report measures (SRM 4)	Self-report measures (SRM 5)	Self-report measures (SRM 6)
McLean Screening Instrument for BPD	X							
12-item Personality Functioning Scale-Brief Form 2.0	X			X	X	X	X	X
Demographics (basic)	X							
Demographics (detailed)		X						
SCID-5-PD BPD section		X						
International Trauma Questionnaire			X					
Adverse Childhood Experiences			X					
Borderline Symptom List-23			X	X	X	X	X	X
Reflective Functioning Questionnaire-8			X		X		X	X
Dissociative Symptoms Scale-Brief			X	X	X	X	X	X
Prodromal Questionnaire-Brief (psychotic symptoms)			X		X		X	X
Disordered eating (Eating Disorder-15)			X		X		X	X
Adult Attention-Deficit Hyperactivity Disorder Scale V.1.1			X					
Autism Spectrum Quotient-10			X					
Medical Outcomes Study Social Support Survey			X		X		X	X
Work and Social Functioning Scale			X		X		X	X
three items from UCLA Loneliness Scale (UCLA-3) and one item recommended by Age UK			X		X		X	X
Everyday Discrimination Scale (Short Version)			X	X	X	X	X	X
Coping Orientation to Problems Experienced Inventory (Brief-COPE)		X		X		X		X
Character strengths (novel measure designed with our LEAP)		X		X	X	X	X	X
Alcohol Use Disorders Identification Test			X	X		X		X
Smoking status (survey questions)			X	X		X		X
Drug use (survey questions)			X	X		X		X
Warwick-Edinburgh Mental Well-being Scale (brief)			X	X	X	X	X	X
Patient Health Questionnaire-9			X	X	X	X	X	X
Generalised Anxiety Disorder			X	X	X	X	X	X
Suicidal Ideation Attributes Scale			X	X	X	X	X	X
Inventory of Statements About Self-Injury			X		X		X	X
Pittsburgh Sleep Quality Index Brief			X	X	X	X	X	X

BPD, borderline personality disorder; LEAP, Lived Experience Advisory Panel; SCID-5-PD, Structured Clinical Interview for Diagnostic and Statistical Manual of Mental Disorders-5 Personality Disorders; UCLA, University of California, Los Angeles.

Baseline interviews are expected to last for approximately 2 hours. It is estimated that the completion of the additional self-report measures will take up to a further 40 min. Cohort recruitment began on 6 October 2025 and will finish by 31 December 2026 with data collection completed by 31 December 2028.

#### Follow-up self-report measures

Participants will complete a similar set of self-report measures every 6 months for up to 30 months (table 1).

#### Sample size

The cohort sample size was informed by the requirements of the two nested substudies: the qualitative interview study, which will recruit a purposive subsample of approximately 40 cohort participants, and the EMA study, which will recruit 100 participants. The overall cohort target of 300 participants was selected to provide sufficient numbers for these nested studies while allowing for attrition and maintaining an adequate sample for the planned longitudinal analyses.

The cohort will recruit, retain and characterise people with complex emotions over 30 months, generating longitudinal data on symptoms and functioning to triangulate with substudy findings and improve understanding of phenotypes, mechanisms and outcomes. A target sample of 300 was selected as a pragmatic recruitment goal, expected to provide a basis for the planned cohort analyses subject to the size and characteristics of the realised dataset.

We assume an approximate retention rate of 80% at each follow-up. Assuming 300 participants at baseline, we will have approximately 240 participants at 6 months and 190 participants at 12 months. Analyses will use all available longitudinal observations under the prespecified missing-data approach rather than requiring complete data at all time points.

#### Analysis

Cohort analyses will comprise descriptive characterisation, exploratory factor analysis (EFA) to identify symptom dimensions and longitudinal modelling of these dimensions, where the realised data support stable and interpretable class solutions.

Baseline and follow-up data will be summarised descriptively to characterise the sample. We will report the proportion meeting DSM-5-TR diagnostic thresholds for BPD,^50^ alongside self-reported indicators of cPTSD, ADHD, autism spectrum disorder (ASD), disordered eating and/or substance misuse disorders, to benchmark our sample and results against previous research. We will examine cross-sectional associations between symptoms, social functioning and histories of interpersonal and other trauma. Recruitment routes will also be described and their associations with symptom, functional and participant characteristics explored.

EFAs will be used to identify symptom dimensions and reduce data complexity before longitudinal trajectory modelling. Factor scores for identified dimensions will be calculated for each participant and used in latent class growth analysis (LCGA)^51^ across three time points. The optimal number of classes will be determined using Bayesian information criterion, entropy, likelihood ratio tests, minimum class size and clinical interpretability. Internal validity will be assessed through associations with independent measures of trauma exposure, psychiatric comorbidity and social functioning not used in class derivation. Stability will be examined through alternative model specifications and bootstrap resampling where feasible.

Missing data patterns will be examined descriptively. Longitudinal models will use full-information maximum likelihood under a missing-at-random assumption, with multiple imputation as a sensitivity analysis where appropriate. Repeated measures will be modelled within the LCGA framework. Recruitment source will be included as a covariate and examined in sensitivity analyses. Longitudinal measurement invariance of multi-item scales will be assessed where appropriate to distinguish true change from changes in measurement properties. Analyses will be interpreted as estimates of association, not causation.

The feasibility and stability of LCGA models will be assessed according to the realised longitudinal sample size, completeness of follow-up and distribution of observations before class enumeration is undertaken. Depending on data availability, alternative approaches will also be explored, including the three-step method,^52^ which uses longitudinal item-level data in LCGA to maximise statistical power while accounting for classification uncertainty.

Sensitivity analyses will compare approaches to missing data, including full-information maximum likelihood and multiple imputation.^53^ Analyses will be considered within defined analysis families, with interpretation based on effect estimates, CIs, model fit and stability, supported by sensitivity analyses rather than nominal p-values. Where these data are insufficient to support a stable and interpretable class solution, continuous growth modelling or descriptive trajectory analyses will be used instead. We will follow best practice for reporting latent trajectory analyses.^54^

#### Ecological momentary assessment quantitative substudy

This substudy will use experiential sampling alongside physiological and behavioural measures captured through consumer wearable devices, such as smartwatches. EMA is well suited to studying complex, dynamic cognitive and behavioural phenomena^55–61^ and has been used in BPD research, with a recent meta-analysis reporting 80% completion rates.^56^ We will:

- Examine networks of association between complex emotions symptoms, processes and symptom/process clusters.
- Quantify temporal instability in relationships, emotions and behaviours using linear and non-linear time series analyses and examine whether changes in stability predict emotional crises.
- Examine associations between self-reported experiences and physiological and behavioural indicators, including heart rate, heart rate variability, physical activity and behaviour patterns.
- Assess the predictive value of continuously measured physiological and behavioural indicators for outcomes associated with complex emotions, including how their importance varies over time and within and between participants. We have used this approach in the past to study bipolar disorder.^57 58^
- Use report-assisted interviews after data collection, presenting participants with summaries of their data and inviting them to ‘think aloud’ about their experiences and interpretation.

#### Participants and sample size

Participants will be recruited from the Hub cohort. A sample of 100 participants providing five assessments per day for 14 days, assuming 75% completion, will provide >90% power to detect small bivariate associations (d=0.2) in a linear mixed-effects model, assuming a conservative intra-class correlation coefficient (ICC) of 0.05.^62^ Planned missingness designs will be considered to reduce participant burden.

Data collection will use the NeuroUX platform, which integrates continuous EMA with physiological and behavioural data from wearable devices and smartphones. The platform is General Data Protection Regulation (GDPR) and Health Insurance Portability and Accountability Act (HIPAA) compliant. Participants will wear a smartwatch to collect heart rate, heart-rate variability, physical activity and sleep data. These data will be combined with experiential assessments of up to 20 items using visual analogue, Likert or multiple-choice scales. The item set was developed and refined with LEAP to maximise acceptability and minimise burden. The first daily assessment will include three sleep questions and the final assessment an item on crises or extreme events that day.

EMA onboarding by a member of the research team will take 30–40 min. Completion of the prompted assessments should take no more than 5 min, although evening assessments may take longer if participants choose to complete the optional free-text journal. Total time spent on EMA assessments will be no more than 35 min per day.

30 participants will be invited to repeat data collection after 6 months to assess temporal stability. A further 30 will participate in report-assisted (‘think aloud’) interviews to understand how they interpret the data and relate these to events associated with experiences of complex emotions.

Recruitment to the EMA study is expected to begin in November 2026 and data collection will be completed by 30 June 2027.

#### Analysis

Principal EMA analyses will examine associations between self-reported emotional experiences and physiological and behavioural measures, within-person temporal variability and temporal associations, and network structure. We will use linear mixed-effects models to test prespecified bivariate associations. In addition, we will use Gaussian graphical^62^ models to produce both within-person and between-person networks of association.^59^ We have used this approach in studying Long Covid, another highly heterogeneous condition.^60^

We will use idiographic network analyses to produce individual patient reports. We will use statistical clustering to group participants according to their individual data summaries. These findings will be used to further iterate our system map.

Our examination of the interaction of emotions, behaviours and physiological data will be exploratory, particularly in relation to the prediction of crises, impulsive behaviours and self-injury. We will use predictive modelling based on machine learning methods, such as random forests or recurrent neural networks to understand which, or a combination of, collected data are most predictive. Predictive modelling and participant-level clustering will be treated as exploratory and undertaken only where the characteristics and completeness of the data support meaningful and stable results. Alternative model specifications will be assessed using prespecified validation and sensitivity procedures. Given the discovery-focused nature of the study, analyses will be considered within defined analysis families, with interpretation based primarily on effect estimates, CIs, model stability and sensitivity analyses rather than nominal p values.

The sample-size calculation relates to the principal linear mixed-effects analysis and does not imply adequate power for more complex exploratory analyses. We will limit overfitting these using cross-validation, hyperparameter tuning and early stopping. Participant-level cross-validation or train/test separation that does not rely on repeated observations from the same participants will be used where applicable.

#### Depth longitudinal qualitative substudy

Around 40 participants will be invited for one or more in-depth interviews. We will purposively sample for diversity in experiences of complex emotions and engagement with mental health services, including people with and without experiences of diagnosis, treatment or care, and those refused care. We will also seek variation in socio-economic circumstances, age, ethnicity, gender, sexual orientation and long-term physical health conditions. Baseline interview findings will inform sampling to capture diverse difficulties, strengths and coping strategies.

Interviews will be conducted in person or online according to participant preference. We will follow our support and safety plans and take care to avoid retraumatising vulnerable participants, using active consent throughout, with interviewers trained in trauma-informed approaches.^63^ Interviews will be audio-recorded, transcribed verbatim and anonymised.

We will explore how participants understand and navigate their emotional lives, relationships, impulsive behaviours (including self-injury) and sense of self over time, and how these experiences relate to coping, help-seeking and social context. We will explore meaning-making, emotional change or continuity, perceived supports or gaps, personal strengths, meaningful outcomes and the influence of social and service environments.

The first interview will draw on a Biographical Narrative Interpretive Method^64 65^ approach by using an opening framing statement which allows the participant to steer their narrative, and open-ended questions to sensitively elicit life stories.^51^ Reflections among the research team and with the LEAP highlighted the risk of framing interviews along diagnostic criteria (eg, asking about experiences of emotional dysregulation), missing both the strengths that people who experience complex emotions have and narrowing the inquiry to experiences which are well documented. We will draw on existing qualitative research to identify under-researched aspects of complex emotions and engage with the LEAP about less well-understood aspects of their experiences. Optional use of participant-chosen images, objects or artworks is an established qualitative elicitation technique that supports memory, emotion and meaning-making, and enables participants to shape their narratives in ways that may be difficult through words alone.^66^ This has proven helpful in trauma-informed and culturally sensitive research with potentially vulnerable groups.^67^

A subgroup of 10 participants will be invited for follow-up interviews, sampled according to willingness to participate and diversity. We will purposively include younger (<25 years) and older (>50 years) participants to explore life course aspects of complex emotions, including childhood adversity, changing relationships and coping strategies, and shifts in societal attitudes, clinical practice and diagnosis. Initial themes will be explored in greater depth, particularly help-seeking, diagnosis and its evolution over time, treatment and support.

LEAP members will contribute to coding and cointerpretation of findings, supported by bespoke training, individual supervision and regular debriefing. This coproduced approach allows both shared patterns and individual nuance to be retained in interpretation.

Recruitment to longitudinal interviews began on 1 June 2026 and will finish by 31 December 2026, and data collection will be complete by 30 September 2027.

#### Analysis

Data will be analysed using reflexive thematic analysis (RTA) and narrative analysis (NA). RTA^68 69^ will identify shared experiences and coping strategies across participants, while NA^70 71^ will examine how individuals make sense of their experiences over time and within their personal histories and circumstances, considering what is told, how and why.

Analysis will be inductive and iterative, incorporating within-case and cross-case comparisons. NVivo or similar software may support coding, which may also be undertaken manually. Recognising the active and subjective role of researchers in constructing themes and interpretations, we will use reflexive journaling and regular discussion among academic and lived experience peer researchers to support analytical development and interpretation.

#### Integration of findings across work packages

Findings from the cohort and nested quantitative and qualitative substudies, informed by evidence synthesis, will be integrated to develop an overall interpretation of the nature, trajectories and mechanisms of complex emotions. We will examine convergence, complementarity and divergence across methods to identify patterns that are consistent and those that vary by context or level of analysis.

Integration will be iterative. Baseline cohort data will inform purposive sampling for the qualitative substudy to capture different symptom and functional profiles, recruitment settings and social contexts. Quantitative and qualitative findings will be compared using joint displays and case matrices at the levels of phenotypes, longitudinal trajectories and hypothesised mechanisms. Qualitative findings will be used to explain, contextualise, qualify, challenge or extend quantitative interpretations.

Findings will be discussed regularly with the research team and lived experience experts. These discussions will inform hypotheses about mechanisms, trajectories and potentially modifiable processes, with integrated interpretations documented as they develop.

Divergent findings will be treated as informative rather than inconsistencies to resolve. We will assess comparability of datasets and constructs, examine relevant cases and subgroups, and determine whether divergence is systematic. We will consider methodological explanations, including differences in timing, measurement and sampling, alongside substantive explanations such as contextual or developmental effects. Integrated interpretations will then be refined to reflect evidence across study components.

### Data statement

Identifiable personal data will be stored on the University of Sheffield Secure Data Service, within Sheffield’s Secure Data Environment, which is accredited to the NHS Data Security and Protection Toolkit and managed in accordance with ISO27001. All personal data will be handled in accordance with UK GDPR and the Data Protection Act 2018.

Screening, consent and survey data will be collected using Research Electronic Data Capture (REDCap), hosted by the University of Sheffield. Access to identifiable data will be restricted to authorised researchers, with permissions managed and monitored by the research fellow. Data will be encrypted in transit and at rest and managed under University IT security policies.

Data will be de-identified before analysis. Interview recordings will be transcribed and identifying information removed. Participants will be assigned unique study IDs, with the linkage file stored separately and accessible only to authorised team members. Access-controlled folders will further restrict access to identifiable data.

#### Data sharing and access

Anonymised data will be accessible to the research team, including named researchers outside the University of Sheffield, through controlled access to specified folders within the University’s secure infrastructure.

Wider sharing will be subject to optional participant consent. Where provided, anonymised data may be shared with DATAMIND through its Trusted Research Environment (TRE), a secure health research data hub managed by Swansea University. The TRE uses Secure Anonymised Information Linkage (SAIL) and Secure eResearch Platform (SeRP) infrastructure and operates under ISO27001, Cyber Essentials and strict information governance procedures. No identifiable data will enter or leave the platform. Contributors will sign data-sharing agreements and establish an appropriate legal basis for sharing. A technical appendix, statistical code and anonymised datasets will be made available through DATAMIND in due course.

### Support and safety

Given the sensitive nature of the study, participants may experience emotional distress, safeguarding concerns or disclose self-harm or suicidal intent. A trauma-informed and person-centred approach will be adopted.^42 58^ Participation is voluntary, and participants may pause, skip questions or withdraw, including during follow-up and this will be explained during baseline interviews. Researchers will agree signals for breaks, provide opportunities to withdraw without penalty and signpost local and national support services where appropriate.

Study questionnaires and EMA responses are not monitored in real time, and the study is not intended to provide clinical monitoring or emergency/crisis support. Participants are informed in the cohort study participant information sheet (PIS) that questionnaire responses are not monitored in real time. This is also restated when they meet with a researcher for the baseline interview and further reiterated at each 6-monthly follow-up, when participants are invited to complete the repeat measures. Participants are informed in the EMA PIS and during their researcher-led onboarding meeting that their responses will not be monitored in real time and that the EMA platform should not be used to seek help in a crisis. This message is also embedded in EMA questions on suicide/self-injurious behaviour and related thoughts.

We provide links to support information at each participant contact, including in our emails and automated participant invitations and reminders. These include details and contacts of NHS mental health crisis services, national support organisations and local support services at each recruitment site. This information is also available through the study website.

Where distress or risk is identified during direct researcher contact, our Participant Distress, Risk and Support Plan and safeguarding procedures are followed. This plan was co-developed with lived experience and clinical colleagues to guide responses to distress and escalation. Adverse events (AEs) and serious AEs (SAEs) will be recorded and monitored, with related and unexpected SAEs reported to the Research Ethics Committee (REC) and sponsor within 15 days, and to the UKRI Mental Health Platform Director and study advisory board (AB). Safeguarding procedures will prioritise participant safety while adopting a non-judgemental stance towards disclosures such as self-harm, recognising these as common coping strategies among people experiencing complex emotions.

Participants aged 16–17 years will provide their own informed consent where they have capacity to understand the study and its implications. Information will be age-appropriate and clearly explain the sensitive nature of the research and limits of confidentiality. Parental or guardian consent will not be required, although participants may involve a parent, guardian or other trusted adult if they wish. Safeguarding and distress procedures will apply to all participants, with additional consideration of the developmental and safeguarding needs of younger participants. Serious safeguarding concerns will be managed according to the approved Distress, Risk and Support Plan and relevant sponsor and local procedures.

Research staff and peer researchers may also experience emotional burden when engaging with sensitive narratives.^72–74^ Additional complexities arise in coproduced research, where lived-experience researchers may navigate both professional and personal emotional demands.^75^ Role-specific training, regular debriefing and supervision, and lone worker protections for in-person interviews will support researcher well-being.^76^

## Ethics and dissemination

The study (cohort and qualitative substudy) has been approved by the East Midlands (Derby) NHS REC and the Health Research Authority (HRA) (IRAS 353789, REC 25/EM/0128; 4 July 2025). The EMA substudy has been approved by the Cambridge East NHS REC and HRA (IRAS 364085, REC 26/EE/0171; 11 August 2026).

Screening will be undertaken on the basis of implied consent and participation is voluntary. Written informed consent or witnessed verbal consent will be obtained before each subsequent stage, and participants may withdraw at any time, from either a substudy or the study overall. Procedures were co-designed with people with lived experience through the LEAP to promote ethical sensitivity and accessibility. The hub AB will review progress and emerging data. Substudies, protocol amendments and publications will be reviewed by the LEAP and AB.

SCID-5-PD and other assessment results will not be routinely disclosed because assessments are conducted for research, not clinical, purposes. Participant information will make this clear. Where assessments raise immediate concerns, we will encourage or support participants to seek clinical help in accordance with the study support and safety procedures. Participants may request access to their results, in which case they will receive a brief report describing self-identified areas of difficulty and strength, without diagnostic interpretation.

Dissemination will be delivered in genuine partnership between academic researchers, the study LEAP and lived experience coinvestigators, drawing on complementary expertise and networks. We will determine which findings warrant dissemination, identify priority audiences and shape how results are communicated across different contexts, including through creative arts and other forms of expression.

We will use evidence about complex emotions mechanisms derived from the literature, our study findings and lived experience to consider ways in which existing interventions might be personalised, and/or how these might inform novel interventions. This will include considering those aspects of treatment or support that can be delivered digitally or through peer support and/or self-care education.^77 78^

All partners will contribute to peer-reviewed publications and conference presentations, ensuring lived experience perspectives inform analysis, interpretation and communication. This approach is formalised in a coproduced authorship policy.
