# Supplementary material for: Exploring the nature, development and effects of complex emotions in Sheffield and Plymouth, UK: a longitudinal mixed methods study protocol
Source: BMJ Open. 2026 Oct 26;16(10):e117088. doi: 10.1136/bmjopen-2026-117088 (PMC13711524; doi:10.1136/bmjopen-2026-117088)
Supplement: Supplementary file 1 [file bmjopen-2026-117088.reviewer_comments.pdf]

PEER REVIEW HISTORY

BMJ Open publishes all reviews undertaken for accepted manuscripts. Reviewers are asked to complete a checklist review form and are provided with free text boxes to elaborate on their assessment. These free text comments are reproduced below.

ARTICLE DETAILS

Title (Provisional)

Exploring the Nature, Development and Effects of Complex Emotions in Sheffield and Plymouth, UK: A Longitudinal Mixed Methods Study Protocol

Authors

Ohlsen, Sally; Anderson, Dion; Bastounis, Anastasios; Bhui, Kamaldeep; Booth, Andrew; Burton, Christopher D; Byng, Richard; Chico, Timothy James; Couperthwaite, Lisa; Crimlisk, Helen; Dalgleish, Tim; Doyle, Michael; Gravestock, Saffron; Hardwick, Rebecca; Horspool, Michelle; Hullock, Katie; Kendall, Tim; Leaviss, Joanna; Markwick, Tiffany; Osmani, Venet; Pinfold, Vanessa; Robinson, Molly; Sullivan, Georgia; Sutton , Anthea; Thomas, Felicity; Thompson, Elissa Rosina; Wilson, Evette; Weich, Scott

---

VERSION 1 - REVIEW

**Reviewer**	**1**
**Name**	**Cwik, Jan**
**Affiliation**	**Universität zu Köln, Clinical Psychology and Psychotherapy**
**Date**	**01-May-2026**
**COI**	**none**

This protocol addresses an important and clinically relevant topic: the lived experience, trajectories, and correlates of "complex emotions" using a transdiagnostic, mixed-methods, longitudinal design. Key strengths include the strong involvement of people with lived experience, the attempt to move beyond the stigmatising label of borderline personality disorder, and the broad recruitment strategy across clinical, community, third-sector, and probation settings.

However, several methodological aspects require clarification. The central construct of "complex emotions" remains broad, the inclusion criteria rely heavily on BPD/personality-disorder-related instruments, the analysis plan is highly exploratory, and the proposed sample size may be insufficient for the complexity of the planned analyses. I therefore recommend revision to improve transparency, coherence, and interpretability.

Major aspects:

1. The transdiagnostic framing is valuable, but the construct of "complex emotions" is insufficiently defined. The protocol should clarify whether emotional dysregulation, identity disturbance, interpersonal difficulties, impulsivity, and self-harm are conceptualised as one coherent construct or as overlapping domains. Since the screening tools are closely linked to BPD and personality pathology, the authors should explain how the sample differs from a lower-threshold BPD/personality-functioning sample. The screening thresholds and inclusion rules should also be clearly justified.

2. There are inconsistencies between the stated aims and the described methods. The abstract refers to physiological and behavioural measures, but these are not described in the methods or measurement table. The protocol also mentions two nested sub-studies, while only the qualitative sub-study is detailed. In addition, six-monthly assessments are too infrequent to study short-term triggers of crises or self-injury. The authors should either add more intensive longitudinal methods or reframe these aims as analyses of longer-term associations.

3. The broad recruitment strategy is a strength, but it may introduce substantial selection bias. Participants recruited through clinical, probation, homeless, community, and peer networks may differ systematically. The authors should describe how recruitment will be monitored and whether quotas or stratification will be used. Recruitment source should be included as a key descriptive and analytic variable. The limitations of recruiting only in Sheffield and Plymouth and excluding non-English speakers should also be acknowledged more clearly.

4. The planned sample of 300 may be adequate for descriptive analyses but appears limited for the broad range of planned analyses, including clustering, latent class analysis, network analysis, and longitudinal subgroup validation. A formal sample-size justification is needed. The authors should also state expected attrition rates and explain how missing data and loss to follow-up will be handled, especially given the likely vulnerability and instability of parts of the sample.

5. The analysis plan lists many possible methods but lacks a clear analytic sequence. The authors should distinguish primary, secondary, and exploratory analyses. For clustering and subgroup analyses, they should specify how clusters will be derived, selected, validated, and tested for stability. The protocol should also describe how missing data, repeated measures, recruitment source, multiple testing, and measurement invariance will be handled. Causal language should be avoided unless a clear causal framework is specified.

6. The follow-up schedule is unclear: the manuscript refers to up to three years of follow-up, but Table 1 appears to end at Month 30, and incentives are described for up to five follow-ups. This should be corrected. The questionnaire battery is extensive, and the authors should report expected completion time, options for breaks, and strategies to reduce participant

burden. Because suicidality and self-injury are assessed repeatedly, the protocol should specify how acute risk will be monitored and managed, including outside working hours.

7. Using the SCID-5-PD BPD section for benchmarking is useful, but the protocol should specify interviewer training, supervision, and inter-rater reliability procedures. The phrase "probable diagnoses" based on self-report questionnaires may be too strong. Unless diagnostic interviews are used, terms such as "screen-positive" or "self-report indicators of" would be more appropriate. The authors should also address overlap between constructs such as trauma, distress, comorbidity, and functional impairment.

8. The qualitative component is promising and well justified, particularly the trauma-informed and lived-experience-led approach. However, the sampling strategy needs more detail. The authors should explain how the 40 interview participants and the 10 follow-up participants will be selected. They should also clarify how reflexive thematic analysis and narrative analysis will be integrated, and describe procedures for reflexivity, supervision, disagreement, role boundaries, and support for peer researchers.

9. The study is described as mixed-methods, but the integration strategy is not sufficiently specified. The authors should clarify whether the design is convergent, explanatory, exploratory, or integrated across waves. They should also explain how qualitative findings will inform or interpret quantitative results, for example through joint displays, case matrices, or triangulation. Without this, the qualitative and quantitative components risk functioning as parallel rather than integrated studies.

10. The planned living systematic map is potentially useful but underdescribed. The protocol should specify eligibility criteria, databases, search strategy, screening procedures, extraction methods, quality or relevance assessment, update frequency, and registration plans. The authors should also clarify how the map will inform the cohort study without increasing post hoc analytic flexibility.

Minor aspects:

• Correct the inconsistency between "up to three years" of follow-up and the Month 30 endpoint in Table 1.

• Replace "probable diagnoses" with more cautious wording unless diagnostic interviews are used.

• Clarify consent and safeguarding procedures for participants aged 16–17.

• Briefly describe anti-fraud procedures for online screening and incentive payments.

• Check the term "reflective thematic analysis"; "reflexive thematic analysis" may be intended.

• State whether SCID-5-PD interviewers are blinded to screening and self-report data.

• Provide more detail on anonymisation and controlled data access, given the sensitivity of the data.

VERSION 1 - AUTHOR RESPONSE

Response to Reviewers' comments

We are grateful for the opportunity to respond to the Reviewer's comments, and we have taken this opportunity to reflect further on this manuscript.

The submitted manuscript described much of the research that is to be undertaken as part of the UKRI Complex Emotions Hub. At the time of our original submission, we had secured ethical approval for most but not all elements of the study. This has now changed and we have secured NHS research ethics approval for the ecological momentary analysis (EMA) sub-study work package.

We had initially intended to include the EMA work package in our manuscript, and in fact did so in our first submission, which was declined on the grounds that not all aspects of the project had secured ethical approval. We therefore removed the EMA study from the re-submission (ie the version that the Reviewer has commented on). On reflection, we feel that this contributed to some of the shortcomings that the reviewer identified.

We would like to include the EMA work package in the manuscript. We believe that this will strengthen the paper and help us to respond to the Reviewer, as it allows us to present the Hub research in its entirety. We believe that this provides a more complete and coherent view of the study and allows us to respond to the Reviewer's comments about our analyses more effectively.

We have made extensive revisions to the manuscript. While responses to the Reviewer's comments required the introduction of substantial sections of new text, we have made further changes to minimise the overall word count.

Our responses to the comments raised by the Reviewer are as follows. Direction to page numbers in the text refer to the version of the manuscript with **Track changes**.

1. *The transdiagnostic framing is valuable, but the construct of "complex emotions" is insufficiently defined. The protocol should clarify whether emotional dysregulation, identity disturbance, interpersonal difficulties, impulsivity, and self-harm are conceptualised as one coherent construct or as overlapping domains.*

The decision to avoid a prescriptive definition of complex emotions is deliberate and driven by the evident limitations of current diagnostic constructs and by the discovery-focused nature of our research.

The nosological status and validity Borderline Personality Disorder remain highly contested, as evidenced by the deliberations and outcomes of the respective DSM-5 and ICD-11 revisions. There is consensus, however, that the phenomena which underly this condition

are multiple and complex, and that a dimensional and trait-based approach to further research is indicated. Hence our decision to move beyond diagnostic categories to studying the phenomena that lie below these.

A starting point for our research is, inevitably, the rich evidence base regarding Borderline Personality Disorder (BPD). We are however approaching this issue from a transdiagnostic perspective, and hence the 'complex emotions' terminology. BPD is arguably the most stigmatised of all mental disorders, and avoiding use of this terminology is also important for being able to work collaboratively with lived experience experts, many of whom have been traumatised by their involvement with health and social care services.

In response to the Reviewer's question, we do **not** view the symptoms and difficulties such as emotional dysregulation, identity disturbance, interpersonal difficulties, impulsivity, and self-harm as one coherent construct (ie as a syndrome). Rather, we see these as separate but related and interacting phenomena.

One aim of the research is to understand how these symptoms interact in real time. We will study the dynamic interactions of different symptoms and their relationship to situational triggers. As part of our phenotyping work, we will map and unpack these complex and dynamic interactions, using novel data collection and analysis methods. This is described in our EMA work package (see p10), which we have now returned to the manuscript, and we hope that this makes the description of the research programme clearer.

Finally, it is important to emphasise that this is discovery science rather than hypothesis testing, and therefore exploratory. A key aim is to better understand the mechanisms underlying complex emotion trajectories and much of the value of the research will emerge from the integration of findings across work packages.

2. *Since the screening tools are closely linked to BPD and personality pathology, the authors should explain how the sample differs from a lower-threshold BPD/personality-functioning sample. The screening thresholds and inclusion rules should also be clearly justified.*

Following on from the above, we feel that this may be a slightly moot point. Inevitably, we have used evidence regarding BPD as the starting point for this research, and clearly the component phenomena within our conceptualisation of complex emotions are also included in the syndrome currently referred to as BPD. The difference from previous research is that we are eliciting and studying these component phenomena (and difficulties) individually (and as a complex interacting system) rather than as a syndromal and monolithic diagnosis.

We have chosen two screening measures to determine eligibility for inclusion in our cohort; one of these is a measure of BPD symptoms (which are all complex emotions), the McLean Screening Instrument for BPD (MSI-BPD) and the other a measure of personality functioning, typically covering inter- and intra-personal functioning, the Personality Functioning Scale-Brief Form 2.0 (LPFS-BF-2.0). The choice of these measures is also in keeping with the views of the DSM-5 and ICD-11 committees which both advocated a dimensional approach to the understanding of BPD based on symptoms and functioning.

There was little evidence to guide us on the choice of scoring thresholds. We have therefore done this in a pragmatic and transparent manner and in attempt to include those with less severe but still important and impactful levels of symptoms, within the context of a modest sample size (n=300). We acknowledge that we may not have explained this fully in our original submission, and we have clarified this on p8:

Page 8:
"Cut-off scores were chosen pragmatically to ensure inclusion of those who experience complex emotions but who might not meet diagnostic criteria."

3. *The analysis plan is highly exploratory, and the proposed sample size may be insufficient for the complexity of the planned analyses. I therefore recommend revision to improve transparency, coherence, and interpretability.*

We hope that re-introducing the EMA work package will help to address this concern, as it provides further justification for the study design and sample size.

A key aim of the cohort study is to establish a well-characterised pool of participants from which to recruit to the two sub-studies, namely the EMA study and the in-depth longitudinal qualitative interviews. As we explain on p9, it was for this reason, and within available resources, that we chose a sample size of 300. The collection and analysis of prospective cohort data represents an opportunity to describe the breadth and severity of complex emotions symptoms in a diverse sample (including the prevalence of neurodiversity and drug and alcohol use) and to estimate associations with functioning. We also intend these analyses to capture trajectories of outcome, within the limits imposed by our sample size.

We agree that the plan for analysis of the cohort data in the original paper was insufficiently detailed, and we have addressed this in the text of the paper (p9). We are grateful for the constructive feedback and the opportunity to revise this part of the manuscript.

We acknowledge that our cohort analysis plans are exploratory, and inevitably not all analyses will generate clear or definitive findings; this is in the nature of this type of study. Much depends on the data and its completeness, as well as the sample size. We will however ensure that we triangulate findings across all three of our work packages, and we are confident that these will inform one another and our eventual phenotyping findings.

We have reinstated the EMA work package along with details of data collection and analysis. This sub-study will also employ wearable devices (smartwatches) to capture data on activity as well as physiological parameters. The analysis plan for the EMA data can be found in the revised manuscript.

4. *There are inconsistencies between the stated aims and the described methods. The abstract refers to physiological and behavioural measures, but these are not described in the methods or measurement table. The protocol also mentions two nested sub-studies, while only the qualitative sub-study is detailed. In addition, six-monthly assessments are too infrequent to study short-term triggers of crises or self-injury. The authors should either add*

more intensive longitudinal methods or reframe these aims as analyses of longer-term associations.

We acknowledge the inconsistencies that the Reviewer has (quite rightly) noted in our manuscript. This reflects the fact that we had originally intended to include the EMA sub-study in this paper but were forced to remove it due to a delay in securing ethical approval for this part of the research. This approval is now in place, and we have reinstated this sub-study to the paper.

We hope that the Reviewer (and readers) can now appreciate the entirety of the programme of research and can see how we will achieve our stated aims.

The Reviewer is completely correct in stating that that the cohort data (collected every 6 months) will not tell us much about short-term triggers for crises and episodes in which self-injury occurs. These findings will come from our EMA study, which will collect data using repeated measures on multiple occasions (daily) from participants over a short period of time (14 days).

5. *The broad recruitment strategy is a strength, but it may introduce substantial selection bias. Participants recruited through clinical, probation, homeless, community, and peer networks may differ systematically. The authors should describe how recruitment will be monitored and whether quotas or stratification will be used. Recruitment source should be included as a key descriptive and analytic variable. The limitations of recruiting only in Sheffield and Plymouth and excluding non-English speakers should also be acknowledged more clearly.*

The Reviewer raises important points. The strength of the study which the Reviewer alludes to is also a challenge, of course, in that there are no benchmarks against which to validate the diversity of our sample. This is the first study of its nature that we are aware of, and we cannot know the ethnic, gender, age or socio-economic characteristic of the population from which all adults with complex emotions in our two sites are drawn. We assume that the population from which our sample is drawn will be socio-economically deprived and diverse in ethnicity, but we cannot quantify the extent of this.

We will document where participants heard about the research in the screening survey in an open text box and collect more detailed information about this when they complete the baseline interview. We will describe the routes into the study in our analyses and will consider the possibility that outcomes may vary between these groups (as described in our Analysis plan). We believe that the main difference will be between those who have and have not been successful in accessing treatment for their complex emotions, and we will test for this.

We have amended our Methods section and Analysis plan to reflect this.

Excluding non-English speakers is a limitation, and we agree that we should state this in the manuscript. We have therefore added the following to the Methods section:

Page 8:
"Exclusion of those unable to complete the study assessments in English is a study limitation, reflecting the absence of validated translations of many study measures."

We do not, however, agree that the same applies to limiting recruitment to Sheffield and Plymouth. We have provided a clear rationale for this on pp6-7.

6. *The planned sample of 300 may be adequate for descriptive analyses but appears limited for the broad range of planned analyses, including clustering, latent class analysis, network analysis, and longitudinal subgroup validation. A formal sample-size justification is needed. The authors should also state expected attrition rates and explain how missing data and loss to follow-up will be handled, especially given the likely vulnerability and instability of parts of the sample.*

We hope that we have addressed many of these concerns above and note that the cohort sample size was largely driven by the requirements of the two main sub-studies. We acknowledge that our sample size is likely to be modest in respect of some of the proposed cohort analyses, and we accept that some of these may not yield readily interpretable results. We are confident, however, that the cohort data we collect (and the analyses arising from these) will be informative in contributing to our efforts at phenotyping complex emotions, particularly when triangulated with the sub-study findings.

We also acknowledge that without the EMA study text, the programme appeared incomplete. We understand why the Reviewer was concerned about the analysis plan as presented in the previous version of the manuscript.

We also accept that we were remiss in the detail we provided regarding the cohort analyses and the justification for these. We have addressed this by the additional text that can found on p9.

7. *The analysis plan lists many possible methods but lacks a clear analytic sequence. The authors should distinguish primary, secondary, and exploratory analyses. For clustering and subgroup analyses, they should specify how clusters will be derived, selected, validated, and tested for stability. The protocol should also describe how missing data, repeated measures, recruitment source, multiple testing, and measurement invariance will be handled. Causal language should be avoided unless a clear causal framework is specified.*

We agree that our plans for analysing the cohort data needed to be clearer. We have therefore addressed these points through the additional text to be found on p9. The respective analysis plans for the two main sub-studies, including the EMA study, can be found in the relevant parts of the paper.

8. *The follow-up schedule is unclear: the manuscript refers to up to three years of follow-up, but Table 1 appears to end at Month 30, and incentives are described for up to five follow-ups. This should be corrected. The questionnaire battery is extensive, and the authors should report expected completion time, options for breaks, and strategies to reduce participant burden. Because suicidality and self-injury are assessed repeatedly, the protocol should*

specify how acute risk will be monitored and managed, including outside working hours.

Thank you for highlighting these matters, including the inconsistency in our statements. Because recruitment to the cohort will take place over a period of approximately 15 months, participants will be invited to take part in a variable number of follow-up assessments. The largest number of follow-up assessments will be 5 (over a period of 30 months). We have corrected this on p9.

We will strive to minimise participant burden. Follow-up assessments will all be completed by self-report (as will many of the baseline measures). We will ensure that this is discussed with all participants at baseline interview, and all will be advised that they can take breaks whenever and for however long they require. We will also ensure that participants know how to reach out to the research team if they require any support with this.

Our research ethics approval allows for us to make 3 attempts to contact any participant who does not respond to the automated reminder to complete follow-up questionnaires, and we will take the opportunity (where needed) to speak to individuals to provide any support and encouragement required, and to reassure them that the link to the survey will remain open for as long as they require. We have not included this detail in the manuscript due to space constraints.

We describe the support and safety procedures for this research on p14. We believe that these are detailed, clear and concise. We have however added a sentence regarding follow-up assessments on p13, in the ethics section.

9. *Using the SCID-5-PD BPD section for benchmarking is useful, but the protocol should specify interviewer training, supervision, and inter-rater reliability procedures. The phrase "probable diagnoses" based on self-report questionnaires may be too strong. Unless diagnostic interviews are used, terms such as "screen-positive" or "self-report indicators of" would be more appropriate. The authors should also address overlap between constructs such as trauma, distress, comorbidity, and functional impairment.*

(i) We have included a description of our procedures surrounding use of the SCID-5 BPD interviews on p8.

(ii) We accept that terminology is important. We have therefore changed the text in way that the Reviewer suggests (p9, Analysis section).

(iii) We are unclear what the Reviewer means by *"address[ing] overlap between constructs such as trauma, distress, comorbidity, and functional impairment"*. These are distinct (but related) concepts which we believe we have used appropriately throughout the manuscript.

10. *The qualitative component is promising and well justified, particularly the trauma-informed and lived-experience-led approach. However, (i) the sampling strategy needs more detail. The authors should explain how the 40 interview participants and the 10 follow-up participants will be selected. They should also (ii) clarify how reflexive thematic analysis and*

narrative analysis will be integrated, and (iii) describe procedures for reflexivity, supervision, disagreement, role boundaries, and support for peer researchers.

(i) We have addressed sampling issues in extensive revisions to the text on pp11-12.

(ii) As per our response to Point 11 (below), we have added additional text describing how we will integrate findings across work packages.

(iii) We would like to draw the Reviewer's attention to the text on p12, which describes how we will involve and support LEAP members to contribute to this part of the research. This will be done through attendance at regular team meetings and through additional and bespoke training (f needed), supervision and debriefing.

11. *The study is described as mixed methods, but the integration strategy is not sufficiently specified. The authors should clarify whether the design is convergent, explanatory, exploratory, or integrated across waves. They should also explain how qualitative findings will inform or interpret quantitative results, for example through joint displays, case matrices, or triangulation. Without this, the qualitative and quantitative components risk functioning as parallel rather than integrated studies.*

We agree that this was an oversight and we are grateful for the opportunity to improve the paper by addressing this. We also believe that re-introducing the EMA sub-study to the paper will highlight the richness of the data (and findings) that we will integrate across the different work packages.

We have added text on pp12-13 which describes our approach to mixed methods, integrative analyses.

12. *The planned living systematic map is potentially useful but underdescribed. The protocol should specify eligibility criteria, databases, search strategy, screening procedures, extraction methods, quality or relevance assessment, update frequency, and registration plans. The authors should also clarify how the map will inform the cohort study without increasing post hoc analytic flexibility.*

On further reflection, and in the light of resource considerations, we have decided not to pursue the systematic evidence map. We have therefore removed this section from the paper.

Minor aspects
(i) Correct the inconsistency between "up to three years" of follow-up and the Month 30 endpoint in Table 1.

Please see our response to point 8 above. We have made this change.

(ii) Replace "probable diagnoses" with more cautious wording unless diagnostic interviews are used.

Please see our response to point 5 (ii) above. We have made this change.

(iii) Clarify consent and safeguarding procedures for participants aged 16–17.

We thank the Reviewer for highlighting the need to clarify procedures for participants aged 16–17 years.

Participants aged 16 years and over are eligible for this non-interventional study and are able to provide their own informed consent where they have capacity to understand the study and its implications. Information and consent procedures will be adapted to ensure that younger participants can understand the study, including its sensitive content, potential risks and limits of confidentiality. Participants may involve a parent, guardian or other trusted adult if they wish, but this is not a requirement where the young person is able to provide informed consent.

Our safeguarding procedures apply to all participants, with additional attention to the developmental and safeguarding needs of participants aged 16 and 17. The study team will follow the approved Participant Distress, Risk and Support Plan and relevant sponsor/local safeguarding procedures in responding to distress, disclosures or concerns about safety. Confidentiality will be maintained wherever possible, but its limits will be explained during consent, including circumstances in which information may need to be shared to protect a participant or another person from serious harm. We have clarified these procedures in the revised protocol.

We have added text on p14 to explain and clarify these matters.

(iv) Briefly describe anti-fraud procedures for online screening and incentive payments.

To prevent fraud, we will check the IP address of each sign up, to ensure they are in the UK and in the Sheffield or Plymouth region. We will make allowance for participants who notify us that they are taking part while away from home or using a VPN but will first confirm these details. The baseline interview, (following screening and consent) must be completed either face to face or online, using video.

If a participant passes these checks but we still have concerns (for example if they gave very stereotypical answers to the SCID questions or give inconsistent accounts), we will take this to the study team for further discussion.

We have included these details in the paper (p7).

(v) Check the term "reflective thematic analysis"; "reflexive thematic analysis" may be intended.

Thank you for picking this up. We have clarified and corrected this ambiguity on p12.

(vi) State whether SCID-5-PD interviewers are blinded to screening and self-report data.

SCID-5-PD interviewers are not blinded to screening and self-report data. We have stated this on p8.

(vii) Provide more detail on anonymisation and controlled data access, given the sensitivity of the data.

We have added these details on pp13-14.

VERSION 2 - REVIEW

**Reviewer**	**1**
**Name**	**Cwik, Jan**
**Affiliation**	**Universität zu Köln, Clinical Psychology and Psychotherapy**
**Date**	**18-Aug-2026**
**COI**	

The revised protocol has improved substantially and is now considerably more coherent. The authors have responded constructively to the previous comments and have made meaningful rather than merely editorial changes.

Most importantly, the ecological momentary assessment sub-study has been reinstated following receipt of separate NHS research ethics approval. This resolves the previous mismatch between the stated aims concerning short-term triggers, physiological and behavioural measures, and the methods actually described. The revised programme now includes a longitudinal cohort, an EMA sub-study with wearable data, and longitudinal qualitative interviews. The Abstract and Methods now consistently describe the two nested sub-studies and the intended integration of cohort, qualitative, physiological, behavioral, and EMA data.

The conceptual framing has also improved. The authors now clarify that emotional dysregulation, identity disturbance, interpersonal difficulties, impulsivity and self-harm are not being treated as manifestations of one coherent syndrome, but as separate, related and dynamically interacting phenomena. They also explicitly frame the programme as exploratory and discovery-focused. The screening criteria and deliberately reduced thresholds are now transparent, including MSI-BPD ≥4 and LPFS-BF-2.0 ≥25 to include individuals with clinically relevant difficulties who may not meet conventional diagnostic thresholds.

The cohort analysis plan is more informative than in the previous version. It now describes the sequence from descriptive and cross-sectional analyses to exploratory factor analysis and latent class growth analysis, provides model-selection and validation criteria, and

addresses missing data, repeated measurements, recruitment source, measurement invariance, and causal interpretation. The mixed-methods integration strategy is also substantially clearer, with purposive sampling informed by cohort findings, use of joint displays and case matrices, and explicit attention to convergence, complementarity and divergence across work packages.

Finally, the procedures concerning consent, data security, participant distress, safeguarding, adverse events and support for researchers and lived-experience partners have been strengthened.

I therefore no longer identify a fundamental design problem that would prevent publication of the protocol. However, a small number of methodological clarifications remain.

Remaining aspects for a revision:

1) The protocol states that a sample of 300 is sufficient for the key cohort analyses and anticipates that 250–300 baseline participants will provide sufficiently stable factor structures. The planned analyses nevertheless include exploratory factor analysis followed by latent class growth analysis across three time points, with class selection based on several statistical and clinical criteria.

The response letter itself appropriately acknowledges that the sample may be modest for some analyses and that some findings may not be readily interpretable. However, the revised protocol still does not report an anticipated retention rate or the effective longitudinal sample expected after two or more follow-up waves.

The authors should therefore:

• replace the unqualified statement that is “sufficient” with more conditional wording;

• state the expected retention or attrition assumptions;

• define a minimum acceptable effective sample and minimum class size for LCGA interpretation;

• specify a fallback strategy, such as continuous growth modelling or descriptive trajectory analysis, if the retained sample is insufficient for stable class enumeration.

This would not require changing the study design, but would prevent the protocol from appearing to guarantee analyses that may not be supported by the realised data.

2) The cohort analysis now has a clearer sequence, but it still includes several alternative approaches “depending on data availability.” The EMA component similarly proposes Gaussian graphical and time-series models, individual- and group-level analyses, statistical clustering, random forests and recurrent neural networks.

This breadth is understandable in discovery science, but it creates substantial analytic flexibility. The protocol should identify:

• the principal descriptive and modeling analyses that are prespecified;

• secondary analyses;

• explicitly exploratory, data-dependent analyses;

• how multiple comparisons or model-selection multiplicity will be handled within defined analysis families.

Where formal multiplicity adjustment is not planned, the authors should state that inference will rely primarily on effect estimates, confidence intervals, model stability, and independent replication rather than isolated nominal p-values.

3) The EMA sample-size calculation is clearly reported and supports the detection of a small bivariate association in a linear mixed-effects model, based on 100 participants completing five assessments per day for 14 days with 75% completion. This is a useful improvement.

However, this calculation does not establish adequate power for:

• individual-level temporal networks;

• nonlinear time-series models;

• multiple physiological and behavioural predictors;

• clustering;

• random-forest models;

• recurrent neural networks.

The more complex predictive analyses should therefore be explicitly described as exploratory or feasibility analyses. The protocol should also briefly state how overfitting will be limited—for example through penalisation, nested cross-validation, participant-level train/test separation and restriction of model complexity relative to the effective sample size.

4) The revised protocol now includes a detailed Participant Distress, Risk and Support Plan, permits participants to pause or skip questions, defines safeguarding and adverse-event procedures, and addresses the additional needs of participants aged 16–17.

However, the protocol does not clearly state whether answers submitted through follow-up surveys or EMA—particularly responses concerning suicidal ideation, self-injury or daily crises—will be monitored in real time. It should specify:

• whether questionnaire and EMA responses are actively monitored;

• the frequency and hours of any monitoring;

• what happens when a high-risk response is identified;

• what participants are told to do outside staffed hours;

• that study platforms must not be used as a substitute for emergency or clinical support, where applicable.

This is particularly important because the study repeatedly administers the SIDAS and ISAS and includes an EMA item concerning crises or extreme events.

5) The authors now explain that participants may take breaks and contact the research team for support. The follow-up schedule has also been corrected to a maximum of five follow-up assessments over 30 months.

Nevertheless, the protocol contains an extensive baseline and repeated follow-up battery covering personality functioning, complex-emotion symptoms, dissociation, psychotic experiences, eating, functioning, loneliness, discrimination, coping, substance use, well-being, depression, anxiety, suicidal ideation, self-injury and sleep. The manuscript still does not report the expected completion time.

Please add approximate completion times for:

• screening;

• baseline interview and questionnaires;

• each six-month follow-up;

• each EMA prompt and the total expected daily EMA burden.

These estimates could be based on piloting or informed estimates and would allow readers to evaluate the feasibility of the retention assumptions.

6) The response letter states that the planned living systematic map was removed because of resource considerations. However, the revised manuscript still describes an evidence-synthesis component involving database searches, study selection, an updatable overview, and development of a system map.

The authors should clarify whether this is:

• a formal systematic or scoping review intended for separate publication;

• an internal, iterative evidence-synthesis activity supporting the research programme;

• or a reduced version of the previously proposed living evidence map.

If it is a formal review component, eligibility criteria, independent screening, extraction, appraisal, registration and reporting guidance should be specified more fully. If it is primarily a contextual programme activity, the text should make this clear and avoid language implying a fully systematic living review.

VERSION 2 - AUTHOR RESPONSE

Response to Reviewer's further comments

We are grateful for the many positive comments on the revised manuscript by the Reviewer, and we are pleased that they found this version to be more consistent and

coherent. The extensive changes that we were asked to make were necessary and have undoubtedly improved the paper.

We are pleased that the Reviewer agreed that re-introducing the EMA study has strengthened the paper. We are glad that they found the conceptual framing of the research and the cohort analysis plan to be clearer and more informative.

We are grateful for the opportunity to provide the methodological clarifications asked for. Our responses to the Reviewer's itemised comments are as follows (page numbers refer to the version of the manuscript with **Track changes**):

1. *The protocol states that a sample of 300 is sufficient for the key cohort analyses and anticipates that 250–300 baseline participants will provide sufficiently stable factor structures. The planned analyses nevertheless include exploratory factor analysis followed by latent class growth analysis across three time points, with class selection based on several statistical and clinical criteria.*

The response letter itself appropriately acknowledges that the sample may be modest for some analyses and that some findings may not be readily interpretable. However, the revised protocol still does not report an anticipated retention rate or the effective longitudinal sample expected after two or more follow-up waves.

The authors should therefore:

(i) Replace the unqualified statement that is "sufficient" with more conditional wording.
See p13: *"A target sample of 300 was selected as a pragmatic recruitment goal, expected to provide a basis for the planned cohort analyses, subject to the size and characteristics of the realised dataset."*

(ii) State the expected retention or attrition assumptions.
See p13: *"We assume an approximate retention rate of 80% at each follow-up. Assuming 300 participants at baseline, we will have approximately 240 participants at 6 months and 190 participants at 12 months. Analyses will use all available longitudinal observations under the prespecified missing-data approach rather than requiring complete data at all time points.*

(iii) Define a minimum acceptable effective sample and minimum class size for LCGA interpretation.
It is difficult to specify a minimum sample size for these analyses because LCGA adequacy depends on things such as the number of classes, class separation, number and timing of observations, missingness, and the complexity of the model. We do however agree that a cautious statement is required, and hope that the following will suffice, on p14:

"The feasibility and stability of LCGA models will be assessed according to the realised longitudinal sample size, completeness of follow-up and distribution of observations before class enumeration is undertaken."

***(iv) Specify a fallback strategy**, such as continuous growth modelling or descriptive*

trajectory analysis, if the retained sample is insufficient for stable class enumeration. This would not require changing the study design but would prevent the protocol from appearing to guarantee analyses that may not be supported by the realised data.

Thank you for this very helpful suggestion, which we have incorporated on p14: ***"****Where these data are insufficient to support a stable and interpretable class solution, continuous growth modelling or descriptive trajectory analyses will be used instead."*

2. *The cohort analysis now has a clearer sequence, but it still includes several alternative approaches "depending on data availability." The EMA component similarly proposes Gaussian graphical and time-series models, individual- and group-level analyses, statistical clustering, random forests and recurrent neural networks. This breadth is understandable in discovery science, but it creates substantial analytic flexibility.*

The protocol should identify:

- *The principal descriptive and modelling analyses that are prespecified; secondary analyses;*
- *Explicitly exploratory, data-dependent analyses;*
- *How multiple comparisons or model-selection multiplicity will be handled within defined analysis families.*

Where formal multiplicity adjustment is not planned, the authors should state that inference will rely primarily on effect estimates, confidence intervals, model stability, and independent replication rather than isolated nominal p-values.

We agree that the protocol should distinguish more clearly between the analyses prespecified to address the cohort and EMA research questions, while indicating that more complex exploratory analyses that will be undertaken only where the characteristics and completeness of the data support meaningful and stable results.

Principal cohort analyses will comprise descriptive characterisation, exploratory factor analysis to identify symptom dimensions, and longitudinal modelling of these dimensions, with LCGA used to identify trajectory subgroups where the realised data support stable and interpretable class solutions. Alternative model specifications and additional subgroup or association analyses will be treated as secondary or exploratory and undertaken according to the characteristics and completeness of the data. Analyses will be considered within defined analysis families, with interpretation based primarily on effect estimates, confidence intervals, model fit and stability and sensitivity analyses rather than nominal p-values.

We have added to the text on cohort data analysis on p13 to emphasise these points.

Principal EMA analyses will examine: (i) associations between self-reported emotional experiences and physiological and behavioural measures; (ii) within-person temporal variability and temporal associations between these measures; and

(iii) the network structure of emotional experiences measured repeatedly during the EMA period.

Predictive modelling and participant-level clustering will be treated as exploratory and undertaken only where these are supported by the characteristics and completeness of the data. Model selection will be based on prespecified validation and sensitivity procedures rather than selection of the model producing the most favourable results. Given the discovery-focused nature of the study, we will not apply a blanket multiplicity correction across all EMA analyses. Instead, analyses will be considered within defined analysis families, with interpretation based primarily on effect estimates, confidence intervals, model stability and sensitivity analyses rather than nominal p-values.

We have addressed these issues in respect of the EMA study on p15:

"Principal EMA analyses will examine associations between self-reported emotional experiences and physiological and behavioural measures, within-person temporal variability and temporal associations, and network structure. Predictive modelling and participant-level clustering will be treated as exploratory and undertaken only where the characteristics and completeness of the data support meaningful and stable results. Alternative model specifications will be assessed using prespecified validation and sensitivity procedures. Given the discovery-focused nature of the study, analyses will be considered within defined analysis families, with interpretation based primarily on effect estimates, confidence intervals, model stability and sensitivity analyses rather than isolated nominal p-values."

3. *The EMA sample-size calculation is clearly reported and supports the detection of a small bivariate association in a linear mixed-effects model, based on 100 participants completing five assessments per day for 14 days with 75% completion. This is a useful improvement.*

However, this calculation does not establish adequate power for:
- *individual-level temporal networks;*
- *nonlinear time-series models;*
- *multiple physiological and behavioural predictors;*
- *clustering;*
- *random-forest models;*
- *recurrent neural networks.*

The more complex predictive analyses should therefore be explicitly described as exploratory or feasibility analyses. The protocol should also briefly state how overfitting will be limited—for example through penalisation, nested cross-validation, participant-level train/test separation and restriction of model complexity relative to the effective sample size.

We agree that the existing sample-size calculation supports the principal linear mixed-effects analysis but may lack power for the more complex EMA analyses. We have therefore clarified that network, clustering and machine-learning analyses, including Random Forests and Recurrent Neural Networks, are exploratory and are

not being presented as formally powered analyses. We have added a statement to this effect and about avoiding over-fitting, on p15:

"The sample-size calculation relates to the principal linear mixed-effects analysis and does not imply adequate power for more complex exploratory analyses. We will limit over-fitting these using cross-validation, hyperparameter tuning, and early stopping. Participant-level cross-validation or train/test separation that does not rely on repeated observations from the same participants will be used where applicable."

4. *The revised protocol now includes a detailed Participant Distress, Risk and Support Plan, permits participants to pause or skip questions, defines safeguarding and adverse-event procedures, and addresses the additional needs of participants aged 16–17.*

However, the protocol does not clearly state whether answers submitted through follow-up surveys or EMA - particularly responses concerning suicidal ideation, self-injury or daily crises - will be monitored in real time. It should specify:

- *Whether questionnaire and EMA responses are actively monitored;*
- *The frequency and hours of any monitoring;*
- *What happens when a high-risk response is identified;*
- *What participants are told to do outside staffed hours;*
- *That study platforms must not be used as a substitute for emergency or clinical support, where applicable.*

This is particularly important because the study repeatedly administers the SIDAS and ISAS and includes an EMA item concerning crises or extreme events.

Thank you for raising these points. We have clarified these issues in the paper on p19:

"Study questionnaires and EMA responses are not monitored in real time, and the study is not intended to provide clinical monitoring or emergency/crisis support. Participants are informed in the cohort study Participant Information Sheet (PIS) that questionnaire responses are not monitored in real time. This is also re-stated when they meet with a researcher for the baseline interview and further reiterated at each six-month follow-up, when participants are invited to complete the repeat measures. Participants are informed in the EMA PIS and during their researcher-led onboarding meeting that their responses will not be monitored in real time and that the EMA platform should not be used to seek help in a crisis. This message is also embedded in EMA questions on suicide/self-injurious behaviour and related thoughts.

We provide links to support information at each participant contact, including in our emails and automated participant invitations and reminders. These include details and contacts of NHS mental health crisis services, national support organisations and local support services at each recruitment site. This information is also available through the study website.

Where distress or risk is identified during direct researcher contact, our Participant Distress, Risk and Support Plan and safeguarding procedures are followed."

5. *The authors now explain that participants may take breaks and contact the research team for support. The follow-up schedule has also been corrected to a maximum of five follow-up assessments over 30 months.*

Nevertheless, the protocol contains an extensive baseline and repeated follow-up battery covering personality functioning, complex-emotion symptoms, dissociation, psychotic experiences, eating, functioning, loneliness, discrimination, coping, substance use, well-being, depression, anxiety, suicidal ideation, self-injury and sleep.

The manuscript still does not report the expected completion time. Please add approximate completion times for:

- *Screening*
- *Baseline interview and questionnaires*
- *Each six-month follow-up*
- *Each EMA prompt and the total expected daily EMA burden*

These estimates could be based on piloting or informed estimates and would allow readers to evaluate the feasibility of the retention assumptions.

Based on our early experience of administering the cohort measures and the (team-based) EMA pilot, we can provide the following approximate timings, which we have now included in the appropriate places in the manuscript:

- Screening: approximately 10 minutes.
- Baseline interviews: These interviews are expected to last for approximately 2 hours. It is estimated that the completion of the additional self-report measures will take up to a further 40 minutes.
- Follow-up questionnaires (every 6 months): approximately 30-40 minutes.
- EMA onboarding: approximately 30-40 minutes.
- Individual EMA prompts: These will take no more than 5 minutes to complete. The evening assessment may take longer where participants can choose to complete the optional free-text journal, which would generally take no more than 15 minutes.
- Expected daily EMA burden: 35 minutes maximum.

6. *The response letter states that the planned living systematic map was removed because of resource considerations. However, the revised manuscript still describes an evidence-synthesis component involving database searches, study selection, an updatable overview, and development of a system map.*

The authors should clarify whether this is:

- *A formal systematic or scoping review intended for separate publication*
- *An internal, iterative evidence-synthesis activity supporting the research programme*

- *Or a reduced version of the previously proposed living evidence map.*

If it is a formal review component, eligibility criteria, independent screening, extraction, appraisal, registration and reporting guidance should be specified more fully. If it is primarily a contextual programme activity, the text should make this clear and avoid language implying a fully systematic living review.

We thank the Reviewer for highlighting this ambiguity. The evidence synthesis component has evolved from the living systematic map described in the original protocol. While the original aim remain - to address uncertainties and information needs across the programme by synthesising evidence from aetiological and interventional research, longitudinal studies and qualitative research - we are now undertaking a formal realist review focused on identifying and refining explanations of the mechanisms underlying complex emotional experiences, including the contexts in which these mechanisms operate. We have provided further details of the key methodological elements of the review and removed references to the living systematic map on p6.

The review is a formal methodological component of WP1 rather than a reduced version of the previously proposed living evidence map, and its iterative findings will inform the wider research programme. It will align with and inform the other WPs and support our plans to integrate findings across the programme.
